# Assembly and comparative analysis of the complete mitochondrial genomes of *Pyrus calleryana* Decne. and *Pyrus betulifolia* Bunge

**DOI:** 10.3389/fpls.2026.1906069

**Published:** 2026-09-21

**Authors:** Huan Zhang, Hongliang Ji, Yutong Chi, Xinge Li, Ran Wang, Yingjie Yang, Dingli Li

**Affiliations:** 1 College of Advanced Agriculture and Life Sciences, Weifang University, Weifang, Shandong, China; 2 Qingdao Key Lab of Genetic Improvement and Breeding of Horticultural Plants, Pear Engineering Technology Research Institute, College of Horticulture, Qingdao Agricultural University, Qingdao, China

**Keywords:** codon usage, evolution, mitogenome, *P. betulifolia*, *P. calleryana*, pear, repeats, RNA editing

## Abstract

*Pyrus calleryana* and *Pyrus betulifolia* are wild pear germplasm resources characterized by robust stress resistance and diverse application potentials, serving as prominent pear rootstocks in China. However, their mitochondrial genomes (mitogenomes) remain poorly characterized. This study presents the sequencing, assembly, and comprehensive analysis of the complete mitogenomes of *P. calleryana* and *P. betulifolia*. The mitogenomes of *P. betulifolia* (432,496 bp) and *P. calleryana* (422,567 bp) are circular DNA molecules, with sizes within the known range for *Pyrus* and smaller than those of cultivated pear. Each genome encodes 35 protein-coding genes (PCGs), 3 rRNA genes, and 19–20 tRNA genes, with no core gene loss. The total number of open reading frames (ORFs) is comparable between the two species, each harboring a small set of species-specific ORFs. The mitogenomes of *P. betulifolia* and *P. calleryana* contain 139 and 136 simple sequence repeats (SSRs), as well as 520 and 480 dispersed repeats, respectively, with both genomes featuring 20 tandem repeats. Among the PCGs, histidine is the most abundant amino acid and CAU is the most frequently utilized codon (Relative synonymous codon usage = 1.55), while most PCGs in both species are under purifying selection (Ka/Ks< 1). Whole-genome alignment revealed a single nucleotide polymorphism (SNP) in both the core energy metabolism genes *nad2* and *atp1.* Additionally, 54 mitochondrial plastid DNA fragments and 616 predicted C-to-U RNA-editing sites were identified in both mitogenomes. Collinearity analysis revealed extensive genome rearrangements in the two species. Phylogenetic trees constructed using both *Pyrus*-specific and *Rosaceae*-wide datasets demonstrate that *P. betulifolia* and *P. calleryana* cluster with other *Pyrus* species, showing the closest relationship to *P. ussuriensis* × *P. communis* ‘Zhongai1’, albeit with limited resolution. This study provides high-quality mitogenome assemblies for *P. calleryana* and *P. betulifolia*, revealing coexisting structural variations and highly conserved sequences, and thereby offers a significant genetic resource for the *Rosaceae* family that will facilitate further investigations into the molecular diversity and genetic evolution of wild pears.

## Introduction

Mitochondrion is an important organelle which supply energy for the metabolism processes in cell through respiration (Liberatore et al., 2016). As a semi-autonomous organelle, it possesses a genetic system distinct from the nuclear genome and is typically maternally inherited (Waltz and Giegé, 2020). Unlike the structurally conserved chloroplast (cp) genomes, plant mitogenomes exhibit extensive variation in both size and structure, and possess a suite of distinctive features that distinguish them from mitogenomes of other eukaryotes: a high proportion of non-coding sequences, abundant repetitive elements, frequent integration of foreign DNA, and low nucleotide substitution rates (Petersen et al., 2020; Yu et al., 2022). In addition, the expression patterns of functional genes in plant mitochondria are specific, characterized by cis-/trans-splicing of introns, RNA editing, and codon usage bias (Petersen et al., 2020; Yu et al., 2022). These remarkable characteristics make plant mitochondria a powerful model for investigating eukaryotic evolution (Wang et al., 2024; Liu et al., 2023). However, their complex features present significant challenges for assembly, hindering progress in related research. With the advent of third-generation long-read sequencing technologies and diverse assembly tools, obtaining complete mitogenomes has become feasible, advancing the rapid development of plant mitochondrial genomics.

*Rosaceae* contains many economically important fruit trees, such as *apple*, *plum*, *peach*, and *pear*. The mitogenomes of *Rosaceae* are relatively conserved in coding sequences but exhibit rich structural variation, making them important materials for studying maternal inheritance, cytoplasmic male sterility, and organellar genome evolution. In recent years, complete mitogenome sequences have been obtained from several *Rosaceae* genera, such as *Malus* and *Fragaria*, with related studies covering genome characterization of new species (Zhang et al., 2025a), elucidation of structural variation mechanisms (Sun et al., 2022), patterns of gene transfer (Liu et al., 2023), and phylogenetic reconstruction (Wang et al., 2024; Sun et al., 2022; Wang et al., 2023). These findings have provided valuable insights into understanding the evolutionary adaptability and species differentiation within this family. Despite these advances, the publicly available mitogenome resources for *Rosaceae* remain limited (Sun et al., 2022; Zhang et al., 2025a; Liu et al., 2023), which has greatly constrained our research on the evolution of mitogenomes in this family.

Pears (*Pyrus* spp.), an important temperate fruit crop with substantial global economic value (Hong et al., 2021a), belong to the family *Rosaceae*, subfamily *Maloideae* (or *Pomideae*). The genus comprises at least 22 primary species and over 5,000 cultivated varieties, naturally distributed across eastern and western Eurasia (Wu et al., 2018; Monte-Corvo et al., 2001). *Pyrus* species readily hybridize due to the absence of reproductive barriers, resulting in high genetic diversity and complex phylogenetic relationships (Silva et al., 2014). This rich genetic variation underlies the long-term adaptability, stress resistance, and breeding potential of *Pyrus* species (Silva et al., 2014).

*P. calleryana* Decne. and *P. betulifolia* Bunge are major wild pear species native to China (Zhang et al., 2024b). Over long-term domestication and cultivation, they have become indispensable rootstock resources for the modern pear industry or variety enhancement purposes owing to their excellent stress tolerance and grafting compatibility. *P. betulifolia* is predominantly used in northern China because of its strong cold and salt-alkali resistance (Zhang et al., 2024b; Lombard and Westwood, 1987), whereas *P. calleryana* is preferred in southern China due to its tolerance to waterlogging and high humidity (Culley and Hardiman, 2007). Mitochondria are closely associated with plant responses to stresses such as waterlogging, drought, and salinity (Liberatore et al., 2016); And early studies uncovered a link between mitochondrial structure and waterlogging tolerance in pear rootstocks (Tamura et al., 1996). Therefore, differences in stress resistance between these two rootstock species are likely linked to genetic variations in their mitogenomes. Additionally, both species have been utilized as widely planted urban greening trees, such as ‘Bradford’ cultivar of *P. calleryana* (Culley, 2017), and its genetic resources remain an important gene pool for ornamental pear breeding. Furthermore, the overlapping distributions of *P. calleryana* and *P. betulifolia*, coupled with the lack of reproductive isolation, facilitate natural hybridization (Silva et al., 2014). The maternally inherited nature of mitogenomes renders them suitable for resolving intra- and interspecific phylogenetic relationships. Comparative mitogenomic analyses of the two species can clarify their phylogenetic relatedness, identify the maternal donors of hybrid progeny, and trace the direction of gene introgression. Recent research has verified that mitogenomes variations trigger cytoplasmic male sterility (CMS) in *Pyrus*. As a maternally inherited trait, CMS is highly valuable for pear crossbreeding and genetic improvement of rootstocks (Han et al., 2025). These findings collectively highlight the great value of mitogenomes for investigating stress tolerance, origins, and phylogenetic relationships of these two important pear rootstocks.

While previous studies based on nuclear genes (Zheng et al., 2014) and chloroplast genomes (Kim et al., 2024) have partially resolved the phylogenetic relationships of some *Pyrus* species, systematic investigations focusing on the mitogenomes of *P. calleryana* and *P. betulifolia* remain scarce. To date, complete mitogenomes of only nine *Pyrus* species have been deposited in the National Center for Biotechnology Information (NCBI) databases (https://www.ncbi.nlm.nih.gov/). Except for *P. betulifolia* (accession numbers ON478165 and MW080658), all others are cultivated pears or hybrids, such as *P. communis*, *P. × bretschneideri* ‘Yali’, and *P. ussuriensis × P. communis* ‘Zhongai1’. Notably, the mitogenome of *P. betulifolia* with accession number MW080658 was first reported by our research team. Most published *Pyrus* mitogenomes have been characterized only superficially in terms of genome size, gene content, and GC content, whereas systematic analyses of deeper genetic features—such as repeat-mediated rearrangements, MTPTs, and RNA editing—remain largely lacking (Chung et al., 2017; Cheng et al., 2020; Cho et al., 2019). Sun et al. (2022) identified domestication-related deletion variations (*DEL-D*) in six *Pyrus* mitogenomes, providing valuable resources for elucidating rootstock resistance mechanisms and supporting precision breeding. However, their study did not explore core evolutionary features, including codon usage bias, repetitive sequence families, RNA editing sites, or selective pressures. More importantly, the mitogenome of *P. calleryana* has not yet been resolved, leaving a gap in comparative and evolutionary analyses between *P. calleryana* and *P. betulifolia* based on mitochondrial genomic data. In summary, the currently insufficient basic data and limited in-depth research on the *Pyrus* mitogenomes restrict understanding of their evolutionary mechanisms, and significantly hinder exploration into the formation mechanisms of maternally inherited genetic traits in these two important pear rootstocks. In this study, we employed Illumina short-read and Nanopore long-read sequencing technologies to assemble high-quality complete mitogenomes of *P. calleryana* and *P. betulifolia*. Our objective was to perform a comprehensive analysis of their genomic features and underlying genetic mechanisms, thereby providing a theoretical foundation for the development of molecular markers. We systematically compared and analyzed gene content, GC composition, SNPs, codon usage bias, repeat elements, RNA editing sites, and the incorporation of sequences from the plastid genome between the two mitogenomes. Furthermore, collinearity analyses with mitogenomes of closely related species retrieved from the NCBI database were conducted to investigate gene rearrangement events during evolution. Phylogenetic relationships within the *Rosaceae*, particularly among *Pyrus* species, were also inferred based on mitochondrial PCGs. These analyses provide the first complete map of the *P. calleryana* mitogenome, and together with the comparative data from *P. betulifolia*, offer novel insights into the evolution of *Pyrus* mitogenomes and the genetic diversity harbored by wild pear species.

## Materials and methods

### Sample collection, DNA extraction and genome sequencing

Leaf samples of *P. betulifolia* were collected from healthy plants growing in Sizhang Town, Sishui County, Jining City, Shandong Province (35°33’ N, 117°28’ E), China (**Figure 1A**). Simultaneously, leaves of *P. calleryana* were obtained from Lishui City, Zhejiang Province, China (28.60, 119.75) (**Figure 1B**). All fresh young leaves were immediately flash-frozen in liquid nitrogen, transported to the laboratory, and stored at ultra-low temperature (-80°C) until DNA extraction. Total genomic DNA was extracted from each sample using a modified CTAB method (Porebski et al., 1997).

**Figure 1:**
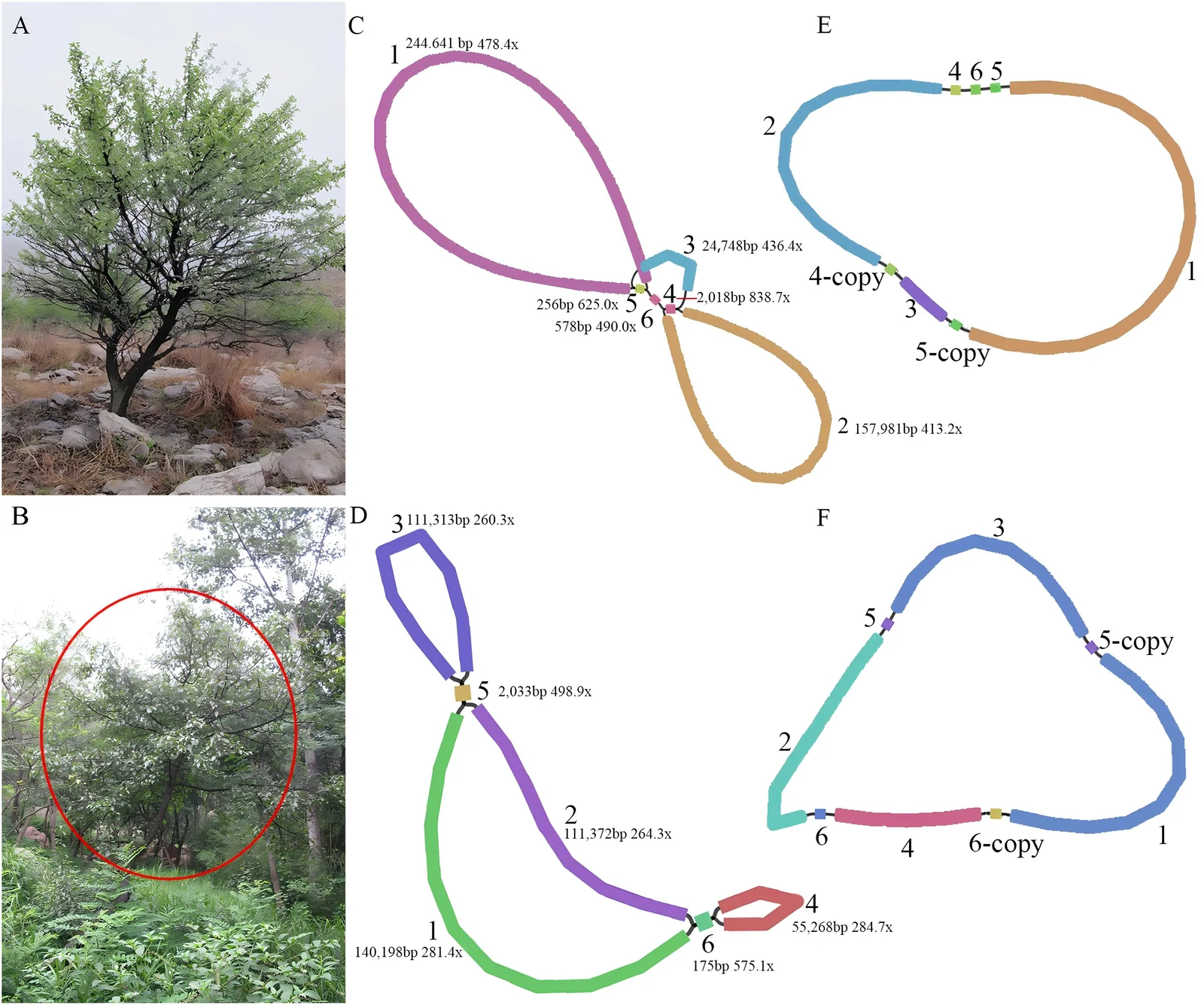
Morphology and mitogenome assemblies of *P. betulifolia* and *P. calleryana*. Whole plants of *P. betulifolia* **(A)** and *P. calleryana* **(B)**. Bandage visualizations of the mitochondrial genome assembly graphs for *P. betulifolia* **(C)** and *P. calleryana* **(D)**, where colored segments represent distinct mitochondrial contigs. Proposed assembly paths leading to the complete linear mitogenomes of *P. betulifolia* **(E)** and *P. calleryana* **(F)**. Different colored segments represent individual contigs, and the color scheme follows the default settings of Bandage software with no specific biological meaning. Numeric labels (e.g., 1, 3, 4) are automatically generated by Bandage to distinguish different contigs. In **(C)**, contig4 and contig5 each occur twice in the corresponding specific solution path **(E)**, and are labeled as ‘4’/’4-copy’ and ‘5’/’5-copy’, respectively. In **(D)**, contig5 and contig6 each occur twice in the corresponding specific solution path **(F)**, and are labeled as ‘5’/’5-copy’ and ‘6’/’6-copy’, respectively.

### Genome sequencing, assembly, and annotation

DNA libraries were constructed and sequenced on the Nanopore PromethION platform (Oxford Nanopore Technologies, Cambridge, UK). Raw Nanopore reads underwent quality control, including filtering and trimming, using NanoFilt and NanoPlot in Nanopack (De Coster et al., 2018). Concurrently, short-read libraries with an average insert size of 350 bp were prepared from high-quality DNA using the Nextera XT DNA Library Preparation Kit (Illumina, San Diego, CA, USA). Sequencing was conducted by Shenzhen Huitong Biotechnology Co., Ltd. (Shenzhen, China) on the Illumina NovaSeq 6000 platform (Illumina, San Diego, CA, USA). Raw Illumina reads were processed with NGS QC Toolkit v2.3.3 (Patel and Jain, 2012) to generate high-accuracy FASTQ data.

The Nanopore sequencing generated 12.35 Gb (read N50: 14.3 kb) and 13.68 Gb (read N50: 10.96 kb) of raw data for *P. calleryana* and *P. betulifolia*, respectively, while the Illumina sequencing produced 10.15 Gb and 14.45 Gb of paired-end reads (2 × 150 bp) for the two species, respectively. The assembly was performed using default parameters unless otherwise specified. Nanopore reads were aligned to reference sequences (*P. ussuriensis* ON478163; *P. betulifolia* ON478165; *P. communis* ON478166) using minimap2 (v2.15-r905), and preliminary assemblies were generated with Miniasm (v0.3-r179) (Li, 2016). Assemblies were polished with NextPolish (v1.3.1) (Hu et al., 2020) using Illumina reads, which were aligned to the polished assemblies with Bowtie2 (v2.3.5.1) using the -very-sensitive-local parameter (Langmead and Salzberg, 2012). The aligned Illumina reads were assembled into contigs using Unicycler (v0.4.8) (Wick et al., 2017), and contig connectivity was visualized with Bandage (Wick et al., 2015). Nanopore reads were re-aligned to contigs, and contig connections were manually verified to obtain complete circular mitogenomes.

Mitogenomes were annotated using Mitochondrial Genome Annotation and Analysis (MITOFY) tool (Alverson et al., 2010) and Mitochondrial Genome Annotation (MFannot) program (Gautheret and Lambert, 2001). Genome maps were visualized using Organellar Genome DRAWing (OGDRAW) tool (Greiner et al., 2019). The assembled sequences have been deposited in NCBI GenBank under accession numbers MW080658 and PX222707.

### ORF prediction and comparative analysis

Open reading frames (ORFs) were predicted using MFannot program and ORFfinder (https://www.ncbi.nlm.nih.gov/orffinder/), with the start codon set to ‘ATG’ and a minimum ORF length of ≥300 bp. Comparative analyses of the mitogenomes of *P. betulifolia* and *P. calleryana* were performed using BLAST (https://blast.ncbi.nlm.nih.gov/Blast.cgi?PROGRAM=blastn&PAGE_TYPE=BlastSearch&LINK_LOC=blasthome).

### Repetitive sequence and homology analysis

SSRs were identified with MISA (v1.0) using default parameters (Beier et al., 2017). Tandem repeats were detected using Tandem Repeats Finder (TRF, version trf409.linux64) with parameters: 2 7 7 80 10 50 2000 -f -d -m (Benson, 1999). Dispersed repeats were analyzed using REPuter with a minimum repeat size of 30 bp, a sequence identity threshold of 90%, and a Hamming distance of 3 (Kurtz et al., 2001).

Chloroplast genomes of *P. betulifolia* (voucher PWH12, GenBank PQ891888.1) and *P. calleryana* (cultivar OPR195, GenBank OK545538.1) were retrieved from NCBI. Homologous fragments between the mitochondrial and chloroplast genomes were identified using BLASTn with an E-value cutoff of 1×10^-5^ (Chen et al., 2015), and visualized using Circos (v0.69) (Zhang et al., 2013).

### RNA editing and codon usage analysis

The PCG sequences of the mitogenome were extracted using a PhyloSuite v1.2.2. RNA editing sites in mitochondrial PCGs were predicted using PmtREP with a cutoff of 0.2 (Mower, 2005). Relative synonymous codon usage (RSCU) values were calculated with CodonW (v1.4.4) (Sharp and Li, 1986), and visualizations were performed in R (v3.6.0).

Codon usage bias (Romero et al., 2000) was further analyzed using EMBOSS-CUSP and CodonW, evaluating parameters such as the effective number of codons (ENC), GC content at the first (GC1), second (GC2), and third (GC3) codon positions, GC content at synonymous third positions (GC3s), and overall GC content (GC-all). ENC plots were generated by plotting observed ENC values against GC3, with standard curves calculated using the formula: $ENC=2+GC_{3}+29/\left[GC3^{2}+2\left(1-GC3\right)\right]$ The ENC ratio was computed as: $ENC_{ratio}=\left(ENC_{theory}-ENC_{practice}\right)/ENC_{theory}$. The frequency distribution of ENC ratios was analyzed to determine factors influencing codon usage bias.

### Ka/Ks and nucleotide diversity analysis

Homologous gene pairs between *P. betulifolia* or *P. calleryana* and 10 related species (species list in **Supplementary Files 1**) were extracted and aligned using ParaAT 2.0 (Zhang et al., 2012). Nonsynonymous (Ka) and synonymous (Ks) substitution rates were calculated using the YN (Yang and Nielsen) method (Yang and Nielsen, 2000) implemented in Ka/Ks_Calculator (v2.0) (Zhang et al., 2006).

To detect positive selection acting on the PCGs, the codeml program implemented in PAML v4.9 was employed to estimate the Ka, Ks, and their ratio (ω). Likelihood ratio tests (LRTs) were performed to compare nested evolutionary models and to identify genes potentially subjected to positive selection. The sequences used for this analysis comprised 29 shared PCGs from the mitogenomes of *P. betulifolia*, *P. calleryana*, and 10 related species (species list in **Supplementary Files 1**).

For each gene, homologous nucleotide sequences were globally aligned using MAFFT v7.313 with the codon alignment mode (Codon Table = 1), while other parameters were kept as default (--auto). The resulting alignments were subsequently trimmed using Gblocks v0.91b to remove poorly aligned regions, with the following parameter settings: Data Type = Codons; Minimum Number of Sequences for a Conserved Position = 7; Minimum Number of Sequences for a Flank Position = 7; Maximum Number of Contiguous Nonconserved Positions = 8; Minimum Length of a Block = 10; Allowed Gap Positions = With Half; and Use Similarity Matrices = Yes.

After trimming, the sequences were converted into PAML-required input format using the Convert Sequence format module implemented in PhyloSuite v1.2.1. Concurrently, a maximum-likelihood phylogenetic tree was constructed using IQ-TREE v3.0.1 under the best-fit nucleotide substitution model, which was subsequently used as the input tree file for downstream analyses.

To estimate ω values for each gene, the branch model implemented in the codeml program of PAML was applied. Specifically, two nested models were compared: (1) the null model (one-ratio model; M0), which assumes a single ω ratio for all branches across the phylogenetic tree; and (2) the branch model (two-ratio model), in which the branch leading to *P. betulifolia* was designated as the foreground branch, while all other branches were treated as background branches, allowing distinct ω ratios for the foreground and background branches. The goodness-of-fit between the two models was evaluated by LRTs. A gene was considered to be under positive selection if the LRT reached statistical significance (*P*< 0.05) and the ω value on the foreground branch was significantly greater than 1.

Multiple sequence alignments of homologous genes were performed with MAFFT (v7.429) (Katoh and Standley, 2013), and nucleotide diversity (Pi) across the mitogenomes was computed in DnaSP (v6.0) using a sliding window of 200 bp and a step size of 100 bp.

### Collinearity and phylogenetic analysis

Pairwise mitogenome comparisons with 10 related Rosaceae species (**Supplementary Files 1**) were performed using BLASTn (E-value ≤1×10^-5^, word size = 7) (Altschul et al., 1990). Homologous regions >1,000 bp were defined as conserved collinear blocks and visualized in multiple synteny plots with TBtools (v2.119) (Chen et al., 2020).

Phylogenetic analyses included *P. betulifolia*, *P. calleryana*, 27 additional *Rosaceae* species (**Supplementary Files 2**), and *Ziziphus jujuba* (PP035764) and *Z. mauritiana* (OP537228) as outgroups. Eleven homologous single-copy genes (*atp4, atp8, ccmFc, ccmFn, cox1, cox2, mttB, nad3, nad4L, nad9, rps3*) were extracted using a Perl script, aligned with MAFFT, and trimmed with Gblocks_0.91b to remove gaps. Trimmed sequences were concatenated and used to construct maximum likelihood (ML) phylogenetic trees with IQ-TREE (v1.6.12) (Nguyen et al., 2015), using the best-fit model K3Pu+F+R2 (Kimura’s three-parameter model with unequal base frequencies and a free-rate model with two rate categories), selected by Bayesian Information Criterion (BIC) and 100 bootstrap replicates for branch support.

To resolve relationships within *Pyrus*, mitogenomes of four *Pyrus* species (*P. × bretschneideri* ‘Yali’, *P. communis*, *P. betulifolia* ON478165.1, and *P. ussuriensis × P. communis* ‘Zhongai1’) were analyzed, with *C. speciosa* OL450370_1 as the outgroup. Twenty-nine shared PCGs (*atp1, atp4, atp8, ccmC, ccmFc, ccmFn, cob, cox1–3, matR, mttB, nad1–6, nad9, rpl10, rpl16, rpl5, rps1, rps3, rps4, rps12, rps13, sdh4*) were extracted, aligned, trimmed, concatenated, and used to construct ML phylogenetic trees following the same pipeline.

## Results

### Features of the *P. betulifolia* and *P. calleryana* mitogenome

The complete mitogenomes of *P. betulifolia* (432,496 bp) and *P. calleryana* (422,567 bp) were successfully assembled through the integration of Illumina short reads and Nanopore long reads, achieving sequencing depths of 224-553× and 287-473×, respectively (**Table 1**, **Supplementary Figure S1**, **S2**). Both mitogenomes exhibited balanced nucleotide compositions (45.2% GC, 54.8% AT) and uniform GC distribution across their circular structures (**Table 1**).

**Table 1:** Detailed information on the nucleotide composition of the whole mitogenome (Total) and its PCGs, tRNAs, rRNAs, Cis-Intron, and IGS in *P.betulifolia* and *P.calleryana.*.

Nucleotide Composition	Gene					
	*P.betulifolia*/*P.calleryana*					
	Total	PCGs	tRNAs	rRNAs	Cis-Intron	IGS
Size (bp)	432496/422567	30234/30234	1488/1412	5138/5138	24117/24109	373559/363714
A + T (%)	54.79/54.77	57.09/57.09	49.6/49.58	48.09/48.09	48.22/48.21	55.11/55.09
G + C (%)	45.21/45.23	42.91/42.91	50.4/50.42	51.91/51.91	51.78/51.79	44.89/44.91

PCGs, Protein-coding genes.

IGS, Intergenic Spacer.

Cis-Intron, A cis-spliced intron removed by cis-splicing within the same RNA molecule.

Visualization of third-generation sequencing data using Bandage revealed complex circular architectures in both species, each comprising six interconnected contigs, the sequence length and sequencing depth of the contigs are shown in **Figures 1C, D**. In **Figure 1C**, the sequencing depths of contig4 and contig5 were approximately twice those of the other contigs, implying that these two contigs may mediate recombination events in the mitogenome of *P. betulifolia* (**Figure 1E**). In **Figure 1D**, the sequencing depths of contig5 and contig6 were about twice those of the other contigs, suggesting that contig5 and contig6 likely mediate recombination in the mitogenome of *P. calleryana* (**Figure 1F**). The specific solution path of *betulifolia* and *P. calleryana* were 1–5–6–4–2-4_copy–3–5_copy (**Figure 1E**) and 1–5_copy–3–5–2–6–4–6_copy (**Figure 1F**), respectively. Here, it is worth noting that the specific solution path displayed in **Figure 1E** and F represents only one plausible structure derived for analytical convenience, and other alternative configurations of the mitogenome are also possible.

To further validate the structural authenticity, we analyzed the alignment of Nanopore long reads across the assembly graphs (**Supplementary Figure S3**-**S6**). The results revealed robust long-read spanning evidence supporting the circularity at the junction points and confirming the physical existence of alternative isoforms (subloops and inversions) mediated by repeats. For *P. calleryana*, where inversion isomers were detected, we selected the dominant conformation supported by the highest depth of spanning long reads as the representative mitogenome (**Supplementary Figure S6**). Importantly, regardless of which structural isoform is ultimately selected, the gene sequence composition and the order of core genes remain identical; thus, the choice of conformation does not affect gene annotation results and subsequent analyses.

Annotation of both mitogenomes revealed a conserved gene set comprising 35 PCGs, 3 ribosomal RNAs (rRNAs), and 19–20 transfer RNAs (tRNAs) (**Figures 2A, B**; **Table 2**), with no core gene losses detected. Strand assignment indicated that 22–23 genes were located on the heavy-strand (H-strand) and 35 genes on the light-strand (L-strand) (**Supplementary Files 3**). Open reading frame (ORF) prediction identified a similar number of ORFs in *P. betulifolia* (109) and *P. calleryana* (104), indicating conserved coding potential, though each species possessed unique ORFs. Seven ORFs (*orf108c, orf111, orf118a, orf119, orf143, orf184*, and *orf99c*) were exclusive to *P. betulifolia*, whereas two (*orf112d* and *orf123a*) were unique to *P. calleryana* (**Supplementary File 4**). Many ORFs were located within introns of known genes (*nad1, nad2, nad4, nad5*, and *nad7*) (**Supplementary File 5**).

**Figure 2:**
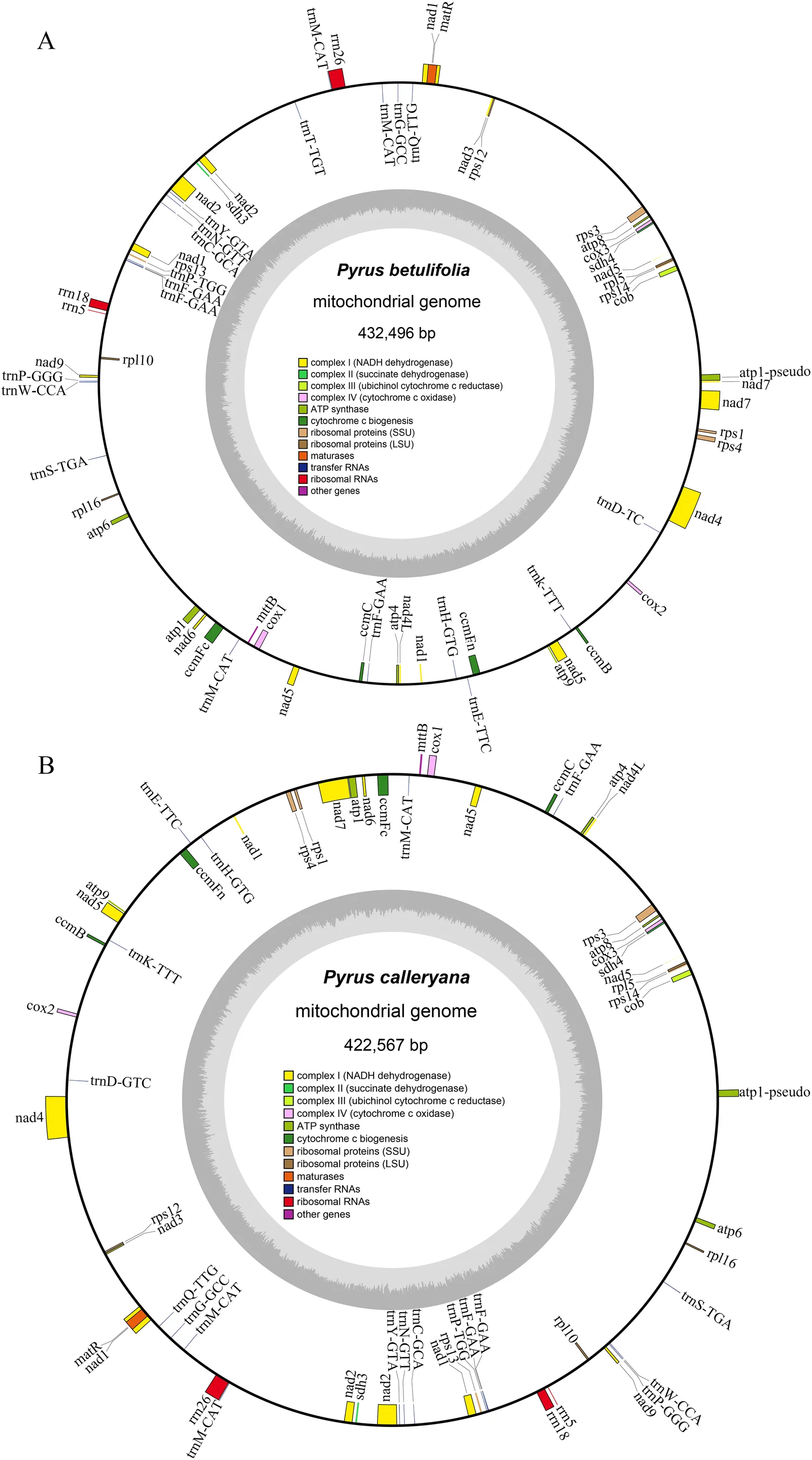
Mitogenome diagrams of *P. betulifolia* **(A)** and *P. calleryana* **(B)**. Genes encoded on the forward strand are shown on the outer circle, whereas those on the reverse strand are placed on the inner circle. The gray ring inside represents the GC content.

**Table 2:** The encoding genes of *P.betulifolia* and *P.calleryana* mitogenome.

Group of genes	Name of genes
ATP synthase	*atp1, atp4, atp6, atp8, atp9*
Cytohrome c biogenesis	*ccmB, ccmC, ccmFc, ccmFn*
Ubichinol cytochrome c reductase	*cob*
Cytochrome c oxidase	*cox1, cox2, cox3*
Maturases	*matR*
Transport membrane protein	*mttB*
NADH dehydrogenase	*nad1, nad2, nad3, nad4, nad4L, nad5, nad6, nad7, nad9*
Ribosomal proteins (LSU)	*rpl10, rpl16, rpl5*
Ribosomal proteins (SSU)	*rps1,rps12, rps13, rps14, rps3, rps4*
Succinate dehydrogenase	*sdh3, sdh4*
rRNAs	*rrn18, rrn26, rrn5*
tRNAs	*trnC-GCA, trnD-GTC, trnE-TTC, trnF-GAA, trnF-GAA, trnF-GAA, trnG-GCC, trnH-GTG, trnK-TTT, trnM-CAT, trnM-CAT, trnM-CAT, trnN-GTT, trnP-GGG, trnP-TGG, trnQ-TTG, trnS-TGA, trnT-TGT* ^*^ *, trnW-CCA, trnY-GTA*

*P. calleryana* lacks a trnT-TGT in its mitochondrial genome compared to *P. betulifolia*.

### Analysis of microsatellites, tandem repeats and dispersed repeat

A total of 139 and 136 SSRs were detected in the mitogenomes of *P. betulifolia* and *P. calleryana*, respectively, ranging from monomers to pentamers; hexamers were absent (**Supplementary File 6**). *P. betulifolia* contained one additional dimer, trimer, and tetramer compared to *P. calleryana*. Mononucleotide repeats were the most abundant, followed by tetramers (**Figure 3A**). Overall, 34 distinct SSR motifs were identified, including 2 mono-, 4 di-, 8 tri-, 18 tetra-, and 2 pentamer motifs (**Supplementary Figure S7a**). The A/T motif predominated, with 47 and 46 occurrences in *P. betulifolia* and *P. calleryana*, respectively, while the AAAG/CTTT motif appeared 16 times in both species (**Supplementary Figure S7a**). Most SSRs were located in intergenic regions (127 in *P. betulifolia* and 124 in *P. calleryana*; **Supplementary Figure S7b**).

**Figure 3:**
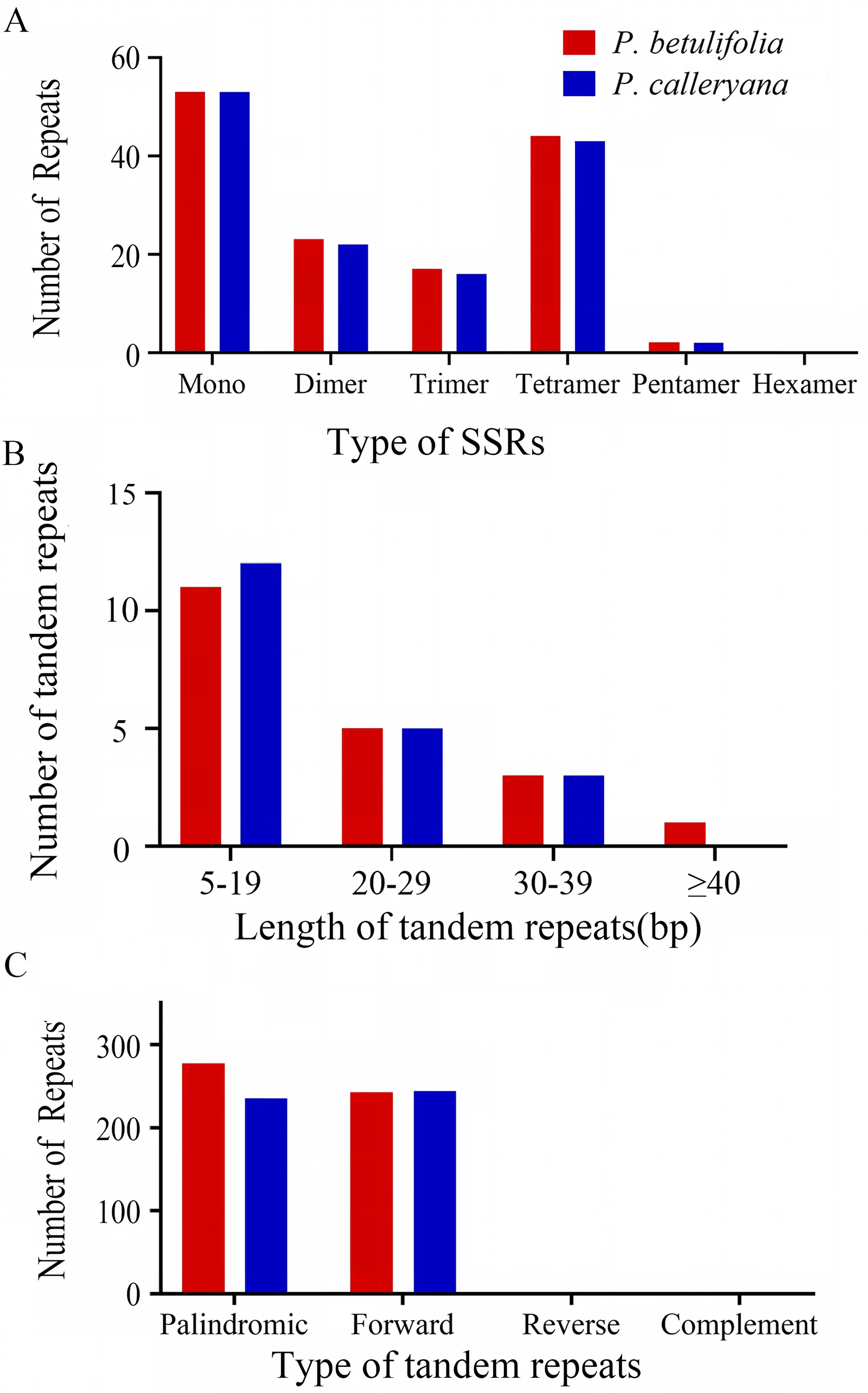
Repeats in the mitogenomes of *P. betulifolia* and *P. calleryana*. **(A)** Types and numbers of SSR repeats. **(B)** Length distribution of tandem repeats. **(C)** Types and numbers of dispersed repeats.

Twenty tandem repeats were identified in each mitogenome (**Supplementary File 7)**. Repeats of 5–19 bp were most frequent (11 in *P. betulifolia* and 12 in *P. calleryana*), followed by 20–29 bp repeats. A 64-bp tandem repeat was unique to *P. betulifolia*, whereas all tandem repeats in *P. calleryana* were shorter than 40 bp (**Figure 3B**).

Dispersed repeat analysis identified 520 repeats in *P. betulifolia* and 480 in *P. calleryana*. In *P. betulifolia*, 242 forward, 277 palindromic, and 1 reverse repeat were annotated, with palindromic types predominating. In *P. calleryana*, 244 forward, 235 palindromic, and 1 reverse repeat were detected, with forward repeats being most abundant (**Supplementary File 8**; **Figure 3C**). Repeats of 30–39 bp were the most numerous (320 in *P. betulifolia* and 300 in *P. calleryana*), followed by 40–49 bp repeats (72 and 60, respectively; **Supplementary Figure S7c**).

### Analysis of chloroplast DNA migration

Analysis of MTPTs identified 54 homologous fragments in each species, totaling 5,209 bp in *P. betulifolia* and 5,201 bp in *P. calleryana*. The longest shared fragment was 847 bp (**Figures 4A, B**). Annotation revealed seven intact tRNA genes (*trnP-UGG*, *trnW-CCA*, *trnH-GUG*, *trnD-GUC*, *trnN-GUU*, *trnM-CAU*, *trnI-CAU/trnM-CAU*) and 13 partial plastid genes (*atpA*, *ndhC*, *psbA*, *psbC*, *psbE*, *rpoC2*, *rps12*, *rrn16S*, *rrn23S*, *trnF-GAA*, *trnP-UGG*, *trnQ-UUG*, and *ycf2*) embedded within the mitochondrial sequences (**Supplementary Files 9**, **10**).

**Figure 4:**
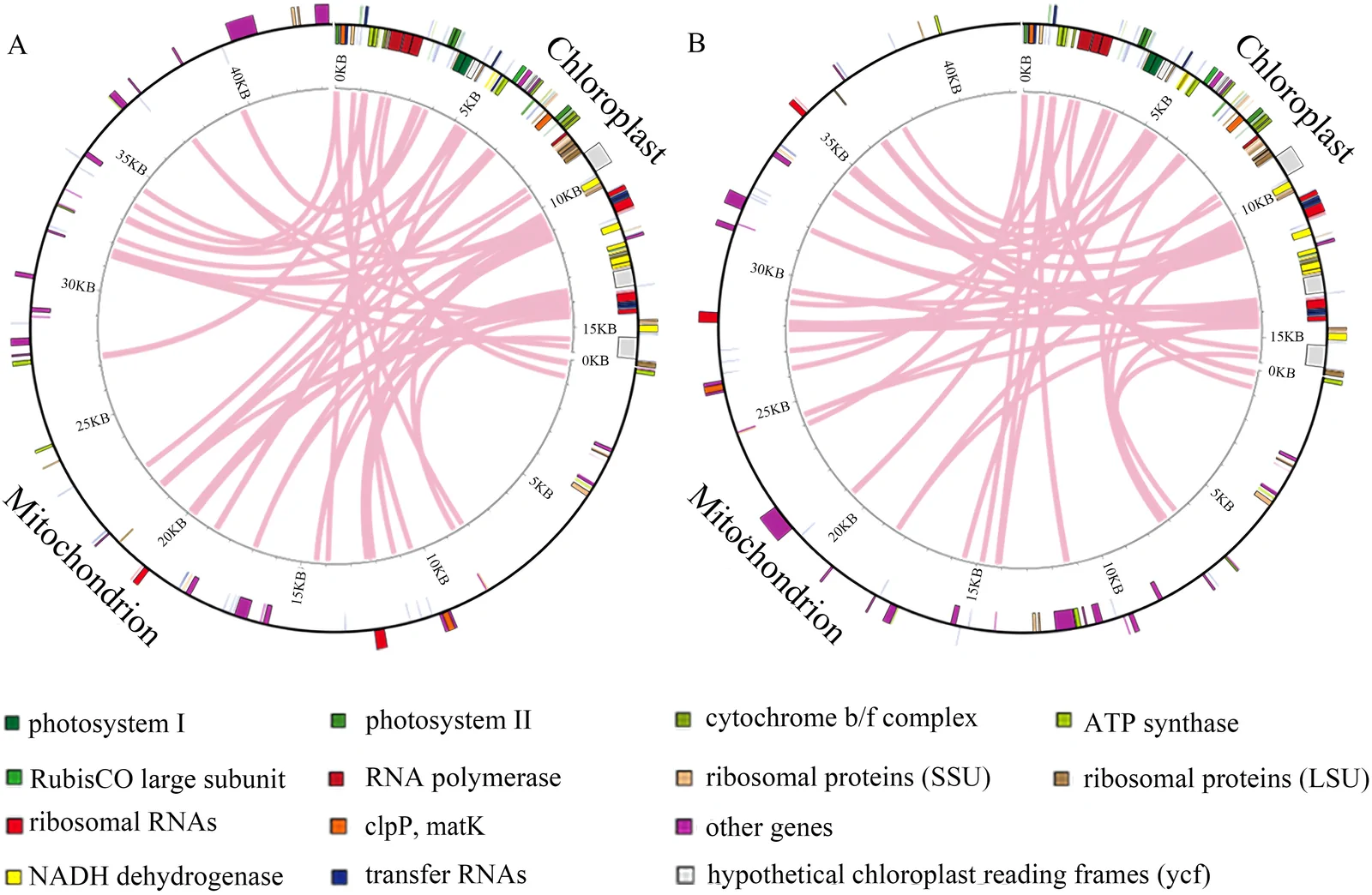
Schematic distribution of homologous sequences between the mitochondrial and chloroplast genomes of *P. betulifolia* **(A)** and *P. calleryana* **(B)**. The outer rings labeled ‘Mitochondrion’ and ‘Chloroplast’ represent the mitochondrial and chloroplast genomes, respectively. A 10-kb genome scale bar is indicated on each ring. The pink arcs in the center denote the 54 identified MTPTs (see**Supplementary Files 9**, **10** for details). Colored ticks on the outer rings mark all annotated functional genes in the mitochondrial or chloroplast genomes.

### Codon usage bias and RNA editing site analysis

Codon usage bias was analyzed for 31 PCGs shared between the mitogenomes of *P. calleryana* and *P. betulifolia* (**Supplementary File 11**; **Figure 5A**). A marked codon usage bias was observed in both species: only the initiation codon AUG, along with GCA (Ala) and UGG (Trp), exhibited RSCU values of 1, whereas 5,546 codons showed RSCU > 1 and were classified as preferentially used. In the high-frequency codon set, adenine (A) or uracil (U) predominated at the third codon position, except for ACC and UUG; conversely, guanine (G) or cytosine (C) occupied the third position in the low-frequency codon set (RSCU< 1), with exceptions including AUA, CUA, and ACA. This pattern is consistent with codon usage observed in terrestrial plant organellar genomes. Histidine codon CAU was the most strongly preferred, with an RSCU of 1.55 in both species.

**Figure 5:**
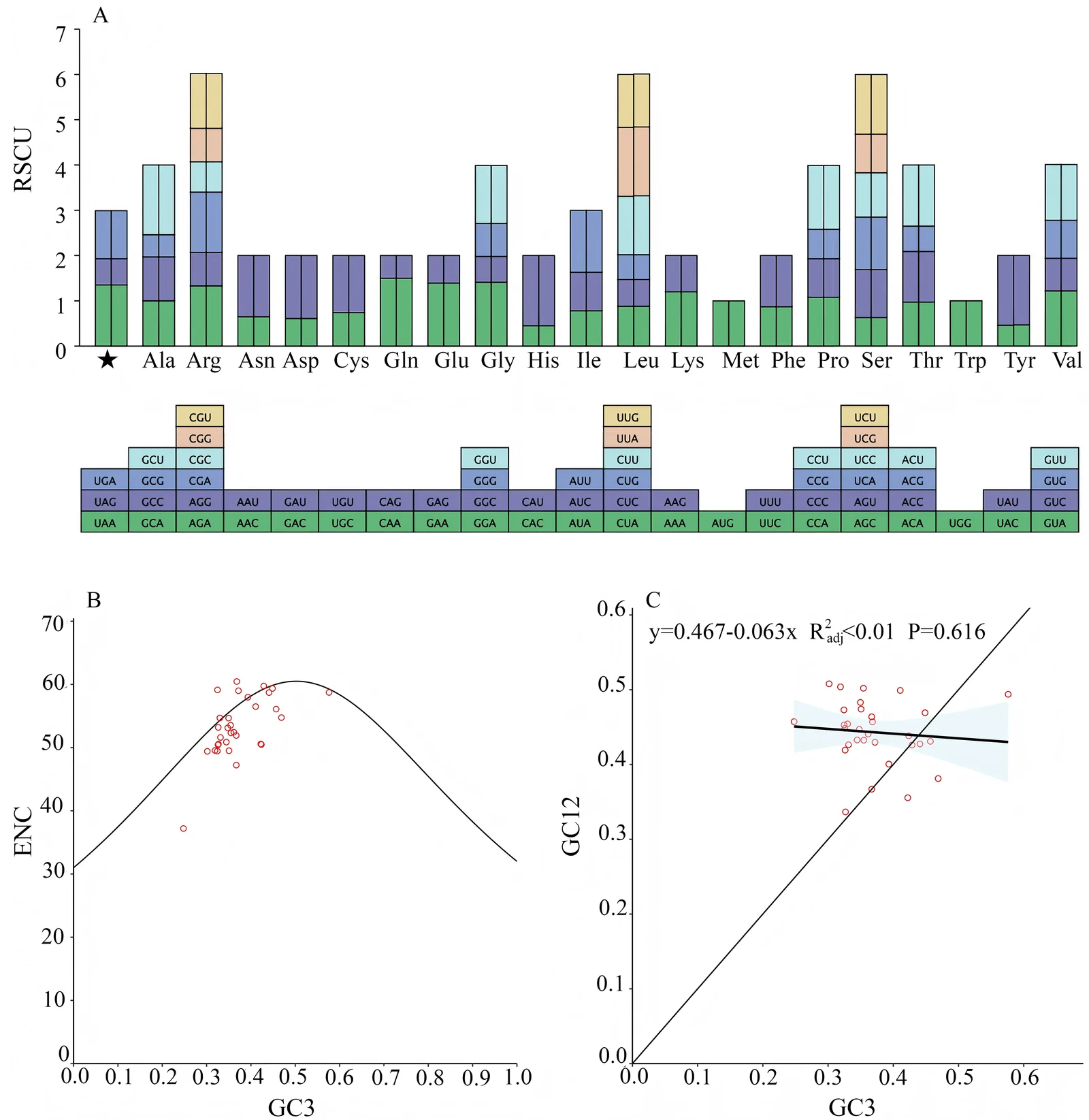
Codon usage bias in the mitochondrial PCGs of *P. betulifolia* and *P. calleryana.* **(A)** RSCU values of mitochondrial PCGs in the two species. The x-axis indicates codon families, and the bar colors correspond to individual codons. **(B)** ENC plot of PCGs in the mitogenomes of *P. betulifolia* and *P. calleryana*. The standard curve was calculated using the equation: ENC = 2+GC3 + 29/[GC3²+(1-GC3)²]. **(C)** Neutrality plot analysis of PCGs in the mitogenomes of *P. betulifolia* and *P. calleryana*. Each dot represents an individual gene, and the black line shows the overall trend.

Analysis of codon base composition revealed similar GC content between the two mitogenomes across codon positions: average GC1 = 47.20%, GC2 = 41.39%, GC3 = 37.30%, and overall GC content ≈ 41.97%. Bases C and G were more frequent at the second codon position (**Supplementary File 12**).

ENC-GC3 plots (**Figure 5B**) showed that most genes were positioned well below the standard curve, suggesting that codon usage bias is primarily shaped by natural selection. A small subset of genes clustered near or above the expected curve, indicating a minor influence of mutation. Further analysis showed that 48% of genes had ENC ratios between 0.05 and 0.15, whereas only 12 genes fell within -0.05 to 0.05, supporting the predominance of natural selection in codon usage patterns.

Neutrality plot analysis (GC12 vs. GC3; **Figure 5C**) yielded low regression slopes (0.0634 in *P. calleryana* and 0.0632 in *P. betulifolia*), reflecting weak correlation between GC3 and GC12. This indicates that mutational pressure had a limited effect on codon usage at the first and second positions relative to the third position, confirming that mutation played only a minor role in shaping codon preference.

RNA editing analysis computationally predicted C-to-U conversions in all 35 mitochondrial PCGs of both species (**Supplementary Figure S8**). A total of 616 predicted RNA-editing sites were identified per species. The *nad4* gene contained the highest number of predicted editing sites, followed by *ccmFn*, whereas *nad4L* harbored the fewest. Notably, no predicted RNA-editing events affected start or stop codons, and all predicted edits resulted in non-synonymous changes. Of the total, 34.12% (211 sites) were predicted to led to leucine incorporation after editing (**Supplementary Files 13**, **14**). All predicted RNA-editing events altered amino acid properties, typically converting residues between hydrophilic and hydrophobic classes.

### Sequence divergence analysis

Pi was calculated for 29 PCGs shared among *P. betulifolia*, *P. calleryana*, and ten closely related species (**Supplementary File 1**). The highest variability was observed in *nad2* (Pi = 0.04667), followed by *sdh4* (Pi = 0.00441) and *atp4* (Pi = 0.00341). Ten genes (*atp9*, *ccmB*, *cox3*, *nad3*, *nad4L*, *nad6*, *nad9*, *rpl5*, *rps12*, and *rps13*) were fully conserved (Pi = 0). All 29 PCGs exhibited Pi< 0.05, highlighting the high sequence conservation of these mitochondrial genes (**Supplementary Figure S9**; **Supplementary File 15**).

Pairwise Ka/Ks analyses were performed separately for *P. betulifolia* and *P. calleryana* against the other 11 species using the same 29 PCGs. Because the two pears differed only at *atp1* and *nad2*, the remaining 27 genes showed identical Ka/Ks patterns. In *P. betulifolia*, Ka/Ks values for *atp1* and *nad2* were consistently< 1 (**Figure 6A**). For *P. calleryana*, Ka/Ks for *atp1* remained< 1 in all comparisons, whereas *nad2* exhibited Ka/Ks > 1 in comparisons with three species, namely *Malus sieversii*, *Photinia serratifolia*, and *Torminalis glaberrima* (**Figure 6B**). It suggesting that the *nad2* gene in *P. calleryana* might have been subjected to positive selection. To validate this finding, a LRT based on the branch model implemented in PAML was further performed on the 29 PCGs across the 12 species to evaluate the selection pressures acting on each gene along this lineage. However, the LRT results showed that the *P*-values for all 29 PCGs were greater than 0.05 (**Supplementary File 16**), failing to reach statistical significance. Consequently, no significant positive selection signal was detected. Therefore, the inference of positive selection acting on the *nad2* gene of *P. calleryana* based solely on Ka/Ks > 1 was not statistically supported.

**Figure 6:**
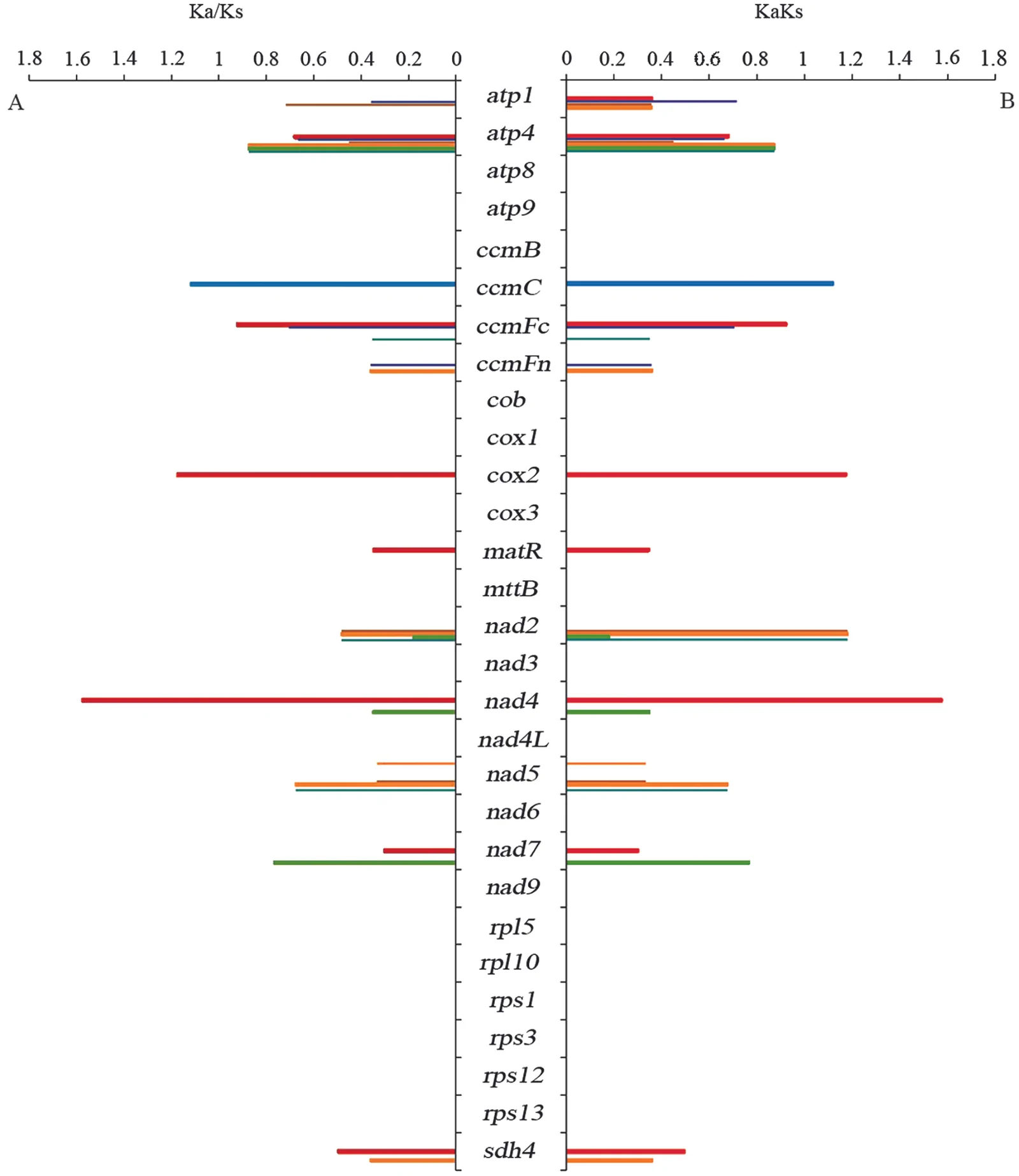
Comparative analysis of Ka/Ks ratios for PCGs. **(A)** *P. betulifolia* compared with 11 other *Rosaceae* species, and **(B)** *P. calleryana* compared with the same 11 *Rosaceae* species.

Collectively, the average Ka/Ks values of the 29 PCGs were all below 1. Taken together with the LRT results, these findings indicate that these PCGs have been predominantly driven by purifying selection during the evolution of *Rosaceae* species, exhibiting a generally conservative evolutionary pattern.

Comparative analysis of the mitogenomes revealed two SNPs in *nad2* and *atp1*. In *nad2*, position 1283 was A in *P. calleryana* and T in *P. betulifolia*; in *atp1*, position 1336 was C in *P. calleryana* and A in *P. betulifolia.* The remaining 33 PCGs were completely conserved between the two species (**Supplementary Files 5**). Protein-level alignment of *nad2* and *atp1* further highlighted amino acid substitutions: in *atp1*, position 446 was isoleucine (I) in *P. calleryana* and leucine (L) in *P. betulifolia*, representing a conservative hydrophobic substitution (I↔L); in *nad2*, position 428 showed a non-conservative substitution from phenylalanine (F) in *P. calleryana* to tyrosine (Y) in *P. betulifolia* (**Supplementary Figure S10**). tRNA comparison showed that *trnM* in *P. calleryana* (77 bp) was three nucleotides longer than in *P. betulifolia*, while the remaining 19 tRNAs and all rRNAs were identical.

### Collinearity and phylogenetic analysis

Collinearity analysis across the 12 compared species identified a total of 245 long collinear blocks with alignment lengths≥1,000 bp (**Figure 7**). Additionally, numerous collinear fragments of less than 1,000 bp were also identified (**Supplementary Figure S11**). Numerous rearranged fragments were detected, and inversions represented the predominant type of rearrangement. These included multiple large-scale rearrangements exceeding 100 kb, with the longest fragment reaching 173,357 bp. The rearranged fragments were highly enriched in core mitochondrial functional genes, especially those associated with energy metabolism and reproduction, including multiple energy metabolism-related genes such as the *cox*, *atp*, *nad*, and *sdh* families. Notably, the longest inversion fragment (173,357 bp) harbored *atp6*, *rpl16*, and *nad9*, while another 74,470 bp inversion fragment contained *mttB* and *ccmFc*. (**Figure 7**; **Supplementary Files 17**-**27**).

**Figure 7:**
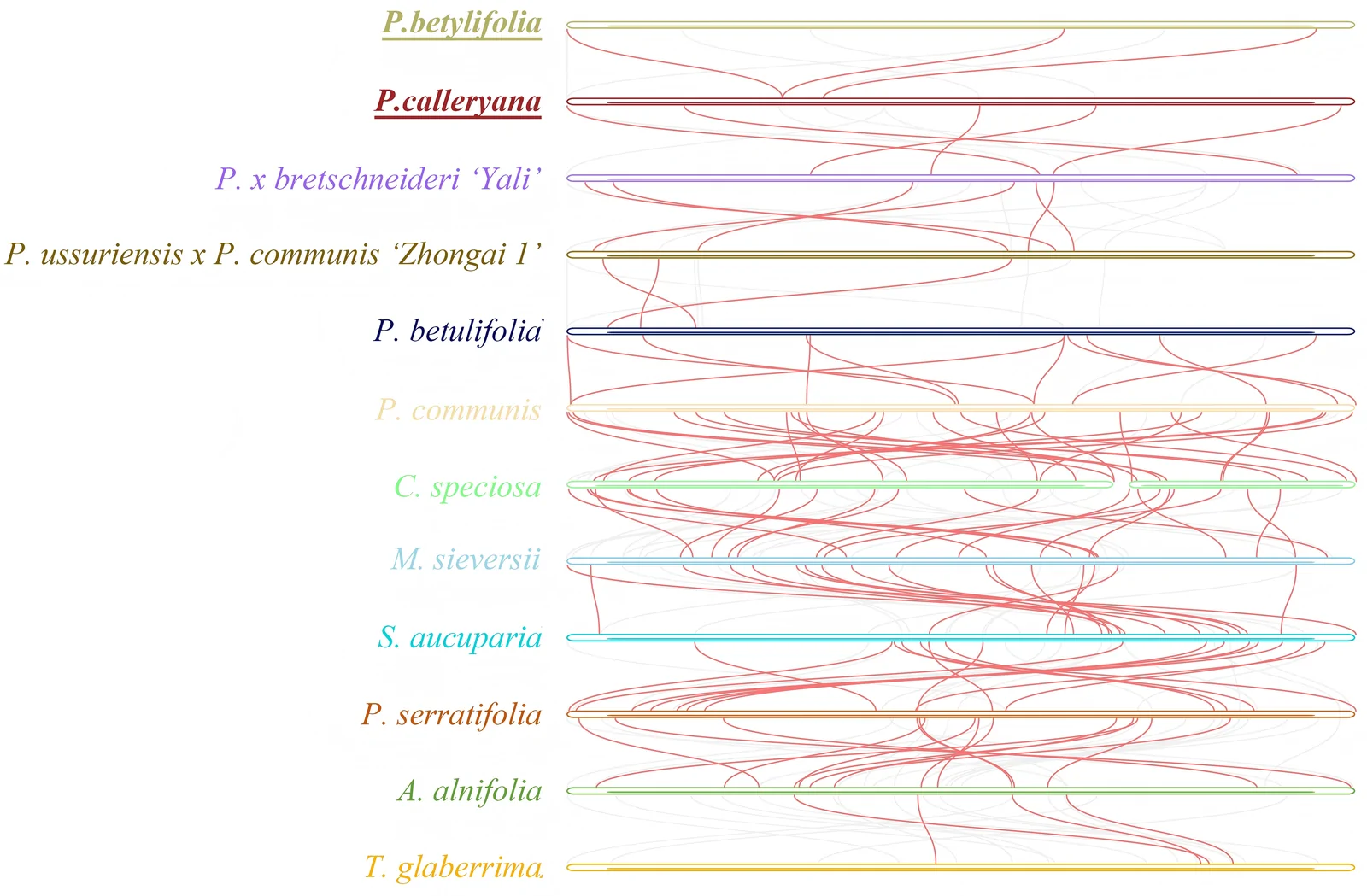
Whole mitogenome collinearity analysis among *P. betulifolia* and *P. calleryana*. Long strips of different colors represent different plant mitochondrial genomes. Liner blocks represent collinear segments.

Phylogenetic relationships were first inferred using 11 homologous single-copy genes from 31 species to determine the evolutionary position of *P. betulifolia* and *P. calleryana* (**Figure 8A**). The outgroups *Ziziphus jujuba* and *Z. mauritiana* (Rhamnaceae) were clearly separated from *Rosaceae* species. Within *Rosaceae*, genera such as *Pyrus*, *Rosa*, and *Photinia* formed distinct clusters. Six *Pyrus* species (*P. betulifolia*, *P. betulifolia* ON478165, *P. ussuriensis × P. communis* ‘Zhongai1’, *P. calleryana*, *P. × bretschneideri* ‘Yali’, and *P. communis*) clustered with *C. speciosa*, indicating a close relationship, though this clade had low bootstrap support (28). Within the *Pyrus* clade, *P. betulifolia* and *P. betulifolia* ON478165 were not grouped together but appeared to form distinct subclades with *P. × bretschneideri* ‘Yali’ and *P. ussuriensis × P. communis* ‘Zhongai1’, respectively. Low bootstrap values (<70%) were observed at multiple nodes within the *Pyrus* lineage.

**Figure 8:**
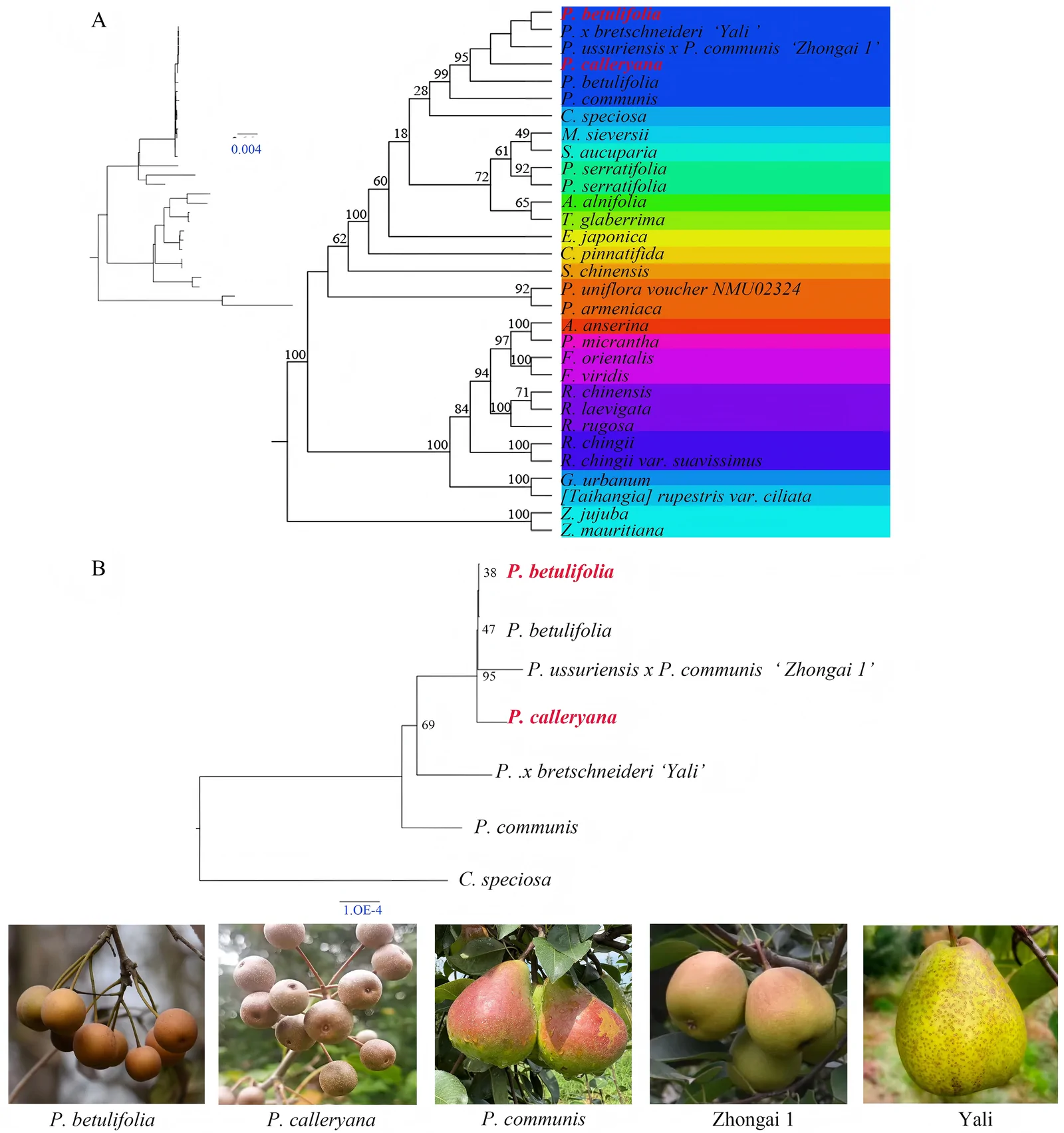
Phylogenetic trees reconstructed from datasets of different species and homologous single-copy genes. **(A)** Phylogenetic tree based on 11 homologous single-copy genes shared among 31 species, with *Z. jujuba* and *Z. mauritiana* used as the outgroup. **(B)** Phylogenetic tree based on 29 homologous single-copy genes shared among 7 species, with *C. speciosa* used as the outgroup. Black numbers indicate bootstrap support values, and blue numbers represent branch lengths.

To further resolve relationships within *Pyrus*, a phylogenetic tree was reconstructed using 29 homologous single-copy genes shared among *P. betulifolia*, *P. calleryana*, four additional *Pyrus* species, and the outgroup *C. speciosa* (**Figure 8B**). All six *Pyrus* taxa formed a distinct clade separated from *C. speciosa*. Within this clade, a subclade was recovered comprising *P. betulifolia*, *P. betulifolia* ON478165, *P. ussuriensis × P. communis* ‘Zhongai1’, and *P. calleryana*, with *P. × bretschneideri* ‘Yali’ as sister to these four species, while *P. communis* was placed on a separate branch. Nevertheless, most nodes within this topology exhibited low support values (<70%).

## Discussion

The mitogenomes structure in *Rosaceae* was highly diverse, with reported architectures including the single circular (Wang et al., 2024), multichromosomal conformations (Zhu et al., 2024), and highly branched (Ha et al., 2025). In this study, the mitogenomes of *P. betulifolia* and *P. calleryana* exhibited a typical single circular structure, consistent with most species of the *Rosaceae* family (Wang et al., 2024; Lu et al., 2023), implying that the structure of mitogenomes may have undergone convergent evolution over evolutionary history. Previous studies indicated that structural divergence of mitogenomes among *Rosaceae* species is largely shaped by the composition of repetitive sequences (Zhu et al., 2024; Ha et al., 2025). In particular, long (>1 kb) and highly homologous repeats are key factors that drive frequent recombination events (Sun et al., 2022) and thereby generate multiple mitochondrial conformations (Zhu et al., 2024; Ha et al., 2025). In the present study, dispersed repetitive sequences in the two pear species were predominantly short fragments of 30–39 bp, and no large repetitive sequences (>1 kb) were detected (**Figure 3**). This likely results in extremely low frequencies of inter- and intramolecular recombination, thus contributing to the structural stability of mitogenomes. Nevertheless, given that the plant mitogenome is not unique and its assembly does not represent a single static form but is instead subject to dynamic changes, we speculate that the mitogenomes of *P. betulifolia* and *P. calleryana* may also exhibit the coexistence of multiple configurations. Future studies should incorporate additional assembly strategies to further validate their structural diversity.

The complete mitogenomes of *P. betulifolia* and *P. calleryana* were determined to be 432,496 bp and 422,567 bp, respectively (**Table 1**), each comprising 35 PCGs, 3 rRNAs, and 19–20 tRNAs. These genome sizes are comparable to previously reported mitogenomes within the *Rosaceae* family, including *Malus* (400,769 bp; GenBank accession NC_065224), and *Sorbus* (455,361 bp; PP506330). Further analysis revealed that the genome sizes of these two wild pear species fall within the known range of *Pyrus* mitogenomes and are highly similar to that of *P. betulifolia* (432,493 bp; ON478165) previously reported by Sun et al. (2022), while being smaller than those of cultivated pear, such as *Pyrus* (443,525 bp; ON478166), *P.bretschneideri* ‘Yali’ (458,895 bp; ON478180), *P. bretschneideri* ‘Dangshansuli’ (458,897 bp; OM763766), *P. communis* (443,525 bp; ON478166). The GC content, a critical indicator of genomic stability and species evaluation, was 45.2% in both species, consistent with values reported in other *Rosaceae* species (43.31–45.62%) (Sun et al., 2022; Lu et al., 2023), suggesting strong evolutionary conservation of GC composition within this family.

Frequent gene loss is a hallmark of plant mitogenomes (Liu et al., 2009). However, in contrast to previous reports on *Pyrus* mitogenomes (Sun et al., 2022), no loss of core PCGs was detected in either species, indicating high conservation of essential mitochondrial functions. Comparative sequence analysis revealed only two SNPs between the two mitogenomes, located in *nad2* and *atp1*, while the remaining 33 PCGs were completely conserved (**Supplementary File 5**). This highlights that although the overall gene composition is highly conserved, limited sequence divergence exists, providing potential species-specific markers for molecular identification.

ORF prediction further corroborated the conserved nature of these mitogenomes, with 109 ORFs in *P. betulifolia* and 104 in *P. calleryana* (**Supplementary File 4**). Notably, each species retained a small set of unique ORFs—seven in *P. betulifolia* and two in *P. calleryana*—which may represent potential candidate markers for distinguishing these two germplasms in future marker development. Collectively, these findings underscore the high conservation of mitochondrial gene content in wild pear species, while revealing limited but informative sequence variation that can facilitate phylogenetic studies and molecular breeding applications. A large number of repetitive sequences, including SSRs, tandem repeats, and dispersed repeats, are frequently observed in plant mitogenomes (Hu et al., 2019; Wynn and Christensen, 2019). Among these, SSRs are highly polymorphic and have become valuable tools for species identification and genetic diversity analysis (Song et al., 2005). Within *Rosaceae*, the number of mitochondrial SSRs varies among species, such as 115 in *Malus* (Wang et al., 2024), 128 in *Sorbus* (Ha et al., 2025), 132 in *P. davidiana* (Zhang et al., 2025a), 112 in *P. pediculata* (Liu et al., 2023), and 52 in raspberry (*Rubus idaeus*) (Zhang et al., 2025b). In this study, 139 and 136 SSRs were identified in the mitogenomes of *P. betulifolia* and *P. calleryana*, respectively, with both genomes predominantly composed of A/T repeats (**Figure 3**, **Supplementary Figure S7a**), consistent with other *Rosaceae* species such as *Malus* (Wang et al., 2024) and *P. pediculata* (Liu et al., 2023). This bias likely reflects the widespread AT preference in plant genomes.

Interestingly, while mono- and dimeric SSRs are typically the most abundant in plant mitogenomes (Song et al., 2021), the second most frequent SSR type in these two pears was tetramer (AAAG/CTTT), rather than dimers (**Supplementary Figure S7a**), a pattern also reported in *P. pediculata* (Liu et al., 2023). This suggests that tetramer enrichment may represent a conserved feature in *Rosaceae* mitogenomes, shaped during SSR evolution. Notably, *P. betulifolia* possessed one additional dimer, one trimer, and one tetramer compared with *P. calleryana*, along with a unique 64-bp tandem repeat (**Figure 3B**), providing a richer set of potential molecular markers for this species.

Dispersed repeats, which are abundant in both species (**Figure 3C**), can influence genomic recombination frequency and reflect evolutionary pressures (Sloan et al., 2012). The presence of these repeats, coupled with observed rearrangements, is consistent with reports in other *Rosaceae* mitogenomes, such as *Malus* (Wang et al., 2024) and *Fragaria* (Fan et al., 2022), highlighting the dynamic and non-conservative structural features of pear mitochondrial genomes.

In this study, dispersed repeats exhibited distinct patterns between the two species. In *P. betulifolia*, 277 palindromic repeats accounted for 53% of the total, whereas in *P. calleryana*, 244 forward repeats were predominant (51%). The prevalence of palindromic repeats suggests their potential role in forming stable secondary structures, which may influence mitochondrial replication or transcriptional regulation (Marechal and Brisson, 2010), implying high structural plasticity in *P. betulifolia* mitogenomes. In contrast, the dominance of forward repeats in *P. calleryana* may reduce recombination frequency, contributing to greater structural stability over evolutionary time. Repeat units of 30–39 bp were the most abundant in both species, representing 62% and 63% of repeats in *P. betulifolia* and *P. calleryana*, respectively. The composition of dispersed repeats in *P. betulifolia* closely resembled that of *Malus* species (Wang et al., 2024). However, Sun et al. (2022) identified a 2040 bp long repeat in *P. betulifolia* with a recombination frequency as high as 77.61-79.37%. Such discrepancy can be attributed to the dynamic nature of plant mitogenomes, which exist as multiple conformational isoforms; additionally, assembly strategies may have limitations in capturing repeat variants with high recombination activity.

Horizontal gene transfer (HGT) from chloroplast to mitochondria is a common feature in plant organelle evolution and contributes to mitogenome size variation (Liu et al., 2023; Hong et al., 2021b). Here, both *P. betulifolia* and *P. calleryana* contained 54 MTPTs totaling 5.2 kb, corresponding to 1.2% of their mitogenomes (**Table 1**, **Figure 4**), indicating highly conserved chloroplast-derived sequence transfer. In angiosperms, MTPTs typically comprise 1–10.3% of mitochondrial genomes (Sloan and Wu, 2014); however, this proportion is generally lower in *Rosaceae*, such as *M. baccata* ‘ZA’ (1.07%) (Wang et al., 2024), *Crataegus pinnatifida* (0.44%) (Zhu et al., 2024), *Rosa* species (3.9%) (Gao et al., 2023), and *P. tenella* (4.14%) (Liu et al., 2024). The 1.2% observed here is consistent with this lower range, supporting the notion that plastid-derived sequences are not a major driver of mitogenome expansion in *Rosaceae* (Putintseva et al., 2020). Several chloroplast-derived tRNA genes, including *trnH-GUG* and *trnN-GUU*, were fully retained, suggesting conserved functionality (Wang et al., 2007). Notably, in contrast to *Aria alnifolia* (Ha et al., 2025) and *P. tenella* (Liu et al., 2024), which retain complete chloroplast PCGs, no intact plastid-derived PCGs were detected in either *P. betulifolia* or *P. calleryana*, highlighting lineage-specific differences in organelle genome integration.

RNA editing is generally recognized as a ubiquitous nucleotide modification process in the organelles of higher plants and is considered to play a role in protein folding and functional regulation (Ichinose and Sugita, 2017). In the present study, using a consistent prediction pipeline, we identified 616 predicted RNA editing sites in both *P. betulifolia* and *P. calleryana* (**Supplementary Figure S8**). It should be noted that the prediction of RNA editing sites is highly susceptible to methodological differences, such as variations in prediction algorithms, the stringency of filtering thresholds, and the completeness of mitogenomes assembly and annotation. These methodological inconsistencies may significantly affect the number and accuracy of predicted editing sites. Indeed, according to independent reports from the original studies, the numbers of mitochondrial RNA editing sites varies among *Rosaceae* species, with 480 sites reported in Chinese plum (Fang et al., 2021), 502 sites in *P. davidiana* (Zhang et al., 2025a), and 262 sites in *P. pedunculata* (Liu et al., 2023). However, due to the aforementioned methodological differences, direct cross-species comparisons based on these published data cannot reliably address the evolutionary dynamics of RNA editing sites among *Rosaceae* species. Future studies should conduct systematic comparative genomic analyses of *Rosaceae* species under unified annotation standards and prediction parameters.

In this study, we predicted RNA editing sites in *P. betulifolia* and *P. calleryana* using the same bioinformatics pipeline, ensuring that the relative differences between these two species are reliable. All predicted sites exhibited C-to-U conversions, consistent with the predominance of this editing type in plant mitogenomes (Liu et al., 2024; Small et al., 2020), and all were predicted to result in non-synonymous codon changes, which may potentially affect protein function (Ichinose and Sugita, 2017). Notably, 34.12% (211 sites) of the predicted edited codons were converted to leucine, a phenomenon also observed in *Astragalus membranaceus* (Zhang et al., 2024a) and *Photinia serratifolia* (Wang et al., 2023). Amino acid property analysis revealed that 19.16% of residues remained hydrophilic post-editing, while 80.84% shifted from hydrophilic to hydrophobic. It has been suggested that such shifts may enhance protein hydrophobicity and structural stability (Zhang et al., 2024a; Wang et al., 2023); however, whether these predicted changes translate into improved mitochondrial protein functionality in *P. betulifolia* and *P. calleryana* requires further investigation. As these sites were obtained via computational prediction, they represent candidate sites for further experimental validation in future studies.

The Ka/Ks ratio is commonly employed to evaluate selective pressures acting on mitochondrial PCGs and to elucidate the underlying evolutionary mechanisms (Zhang et al., 2006). Generally, most PCGs experience neutral or purifying selection due to their highly conserved nature, resulting in Ka/Ks values below 1 (Zhang et al., 2025a). This result has been reported in multiple *Rosaceae* species, such as *P. serratifolia* (Wang et al., 2023). In our analysis of 29 homologous single-copy mitochondrial genes from *P. betulifolia*, *P. calleryana*, and 10 related species, the majority of PCGs were under negative selection (**Figure 6**), reflecting strong conservation across *Rosaceae*.

Collinearity analysis is a valuable approach for exploring phylogenetic relationships and examining conservation and rearrangements of homologous genomic regions (Zhang et al., 2023). Extensive genomic rearrangements have been documented in the mitogenomes of various *Rosaceae* taxa, including *P. davidiana* (Zhang et al., 2025a) and *Chaenomeles speciosa* (Cao et al., 2023). In this study (**Figure 7**), the inconsistent order of collinear blocks in *P. betulifolia* and *P. calleryana* mitogenomes suggests extensive structural reorganization, which may have contributed to the evolution and diversification of the *Pyrus* mitochondrial genome (Christensen, 2013). Such large-scale structural variations are rarely observed in non-hybrid *Pyrus* species, suggesting a close association with interspecific hybridization. These core gene variations might affect hybrid fertility and adaptability, providing functional evidence for the involvement of rearrangements in speciation (Cole et al., 2018). For example, the longest inversion fragment (173,357 bp) harboring *atp6*, *rpl16*, and *nad9* might alter hybrid energy supply efficiency according to this genomic comparison, while inversions containing respiratory chain genes (e.g., *nad2*, *sdh3*) could be speculated to enhance environmental adaptability, though such effects are not experimentally verified. Additionally, the 74,470 bp inversion fragment carrying *mttB* and *ccmFc* may be associated with *Pyrus* pollen fertility, and might hint at the potential role of mitogenomic rearrangements in speciation and adaptive evolution, but this association requires functional validation.

Plant mitogenomes are generally characterized by slow evolutionary rates and highly conserved gene sequences and composition, which limits their utility in phylogenetic studies. Our results further underscore the constraints of using mitochondrial genomes for resolving relationships among closely related taxa. Phylogenetic analysis based on 11 homologous single-copy genes from 31 taxa indicated that six *Pyrus* species cluster with *Chaenomeles speciosa* (**Figure 8A**), but the bootstrap support value was only 28%, reflecting substantial uncertainty in their inferred relationships. Specifically, the evolutionary positions of *P. betulifolia*, *P. betulifolia* ON478165, and *P. calleryana*, all classified as oriental pears, remained unresolved. This ambiguity likely arises from the high sequence similarity among mitochondrial PCGs, limited accumulation of mutations, and insufficient apomorphic characters, which collectively hinder the effective reconstruction of phylogenetic relationships between closely related species. Similar limitations have been reported in *Aria alnifolia* (Ha et al., 2025), *P. serratifolia* (Wang et al., 2023), and *Indocalamus longiauritus* (Liu et al., 2025), reinforcing the challenges of employing mitochondrial PCGs for phylogenetic inference.

After expansion to 29 PCGs (**Figure 8B**), the six *Pyrus* taxa formed a clade. In particular, oriental pears (*P. betulifolia*, *P. betulifolia* ON478165, *P. calleryana*, and *P. ussuriensis* × *P. communis* ‘Zhongai1’) were separated from the western pear (*P. communis*), which is consistent with previous studies based on morphological and genetic evidence (Teng, 2017). This result contrasts with the conclusion of Kim et al. (2024), who placed *P. betulifolia* within the occidental pear clade based on chloroplast genome analysis, suggesting that different cytoplasmic genomes may have followed independent evolutionary trajectories within the genus *Pyrus*. Notably, Sun et al. (2022) also supported the separation of Asian and European pear lineages based on mitochondrial genome data, further reinforcing the utility of mitogenomes for elucidating the maternal evolutionary history of *Pyrus*. Nevertheless, several nodes still exhibit low bootstrap support, indicating that additional high-quality sequences and broader taxon sampling are required to further evaluate the monophyly of *Pyrus* at the mitochondrial genome level.

## Conclusion

In this study, we report high-quality complete mitogenomes of two wild *Pyrus* species, *P. betulifolia* and *P. calleryana*. Both species share a conserved gene complement, including 35 PCGs, three rRNAs, and 19–20 tRNAs, but show substantial structural rearrangements. Only two SNPs were detected between the two species, located in the *nad2* and *atp1* genes. Furthermore, most PCGs are subject to purifying selection, indicating the conservation of mitochondrial genes during the evolution. Species-specific variation in SSRs and tandem repeats may offer valuable resources for developing molecular markers in *Pyrus*, while the conserved presence of MTPTs suggests the co-evolution between plastids and mitochondria. Phylogenetic analyses further indicate that mitogenome-based resolution among *Pyrus* species remains relatively low, likely due to maternal inheritance and a low nucleotide substitution rate, highlighting the limitations of mitochondrial genomes for species delimitation.

## Data Availability

The datasets presented in this study can be found in online repositories. The names of the repository/repositories and accession number(s) can be found in the article/**Supplementary Material**.
