# Supplementary material for: Assembly and comparative analysis of the complete mitochondrial genomes of Pyrus calleryana Decne. and Pyrus betulifolia Bunge
Source: Front Plant Sci. 2026 Sep 21;17:1906069. doi: 10.3389/fpls.2026.1906069 (PMC13640305; doi:10.3389/fpls.2026.1906069)
Supplement: Supplementary file 1 [file Table15.docx]

**Fig. S1 Coverage depth distribution for the mitogenome of *P. betulifolia*.** The estimated mean coverage depth is 224.16× based on second-generation sequencing data (A) and 472.91× based on Nanopore long-read sequencing data (B).

**Fig.S2 Coverage depth distribution for the mitogenome of *P. calleryana*.** The estimated mean coverage depth is 553.02x based on second-generation sequencing data (A) and 287.49× based on Nanopore long-read sequencing data (B).

**Fig.S3 Long-read coverage and structural variation graph for *P. betulifolia***. The alignment of third-generation sequencing reads against the linearized mitogenome is shown. Off-diagonal signals and clusters indicate the presence of alternative recombinant conformations mediated by repeat sequences.

**Fig.S4 Assembly path selection for *P. betulifolia* based on long-read spanning evidence.** The connecting lines represent the long Nanopore reads bridging the repeat regions, supporting the chosen extraction path for the complete circular mitogenome.

**Fig.S5 Long-read coverage and structural variation graph for *P. calleryana*.** Off-diagonal dot clusters represent structural variations (recombination events) resulting from repeat sequences, validating the dynamic nature of the mitogenome.

**Fig.S6 Assembly path selection for *P. calleryana* based on dominant long-read spanning evidence.** Among the detected inversion isomers, the specific conformation supported by the highest depth of bridging Nanopore reads was selected as the representative genome.

****Fig.S7 Distribution characteristics of repetitive elements in the**** mitogenomes ****of** *P. betulifolia* **and** *P. calleryana***.** (A) Abundance distribution of different SSR motif types in the two** *Pyrus* **species. (B) Distribution of SSR repeat frequencies within CDS, IGS, and introns. (C) Length distribution of dispersed repeats.**

**Fig.S8 The distribution of RNA editing sites among PCGs in the *P. betulifolia* and *P. calleryana* mitogenome.**

**Fig.S9 Nucleotide diversity analysis of 12 *Rosaceae* species.**

**Fig.S10 Sequence alignment of the proteins encoded by the *atp1* and *nad2* genes in *P. betulifolia* and *P. calleryana*.** Numbers on the right indicate amino acid residue positions; identical residues are shown with conserved characters, while variable residues are displayed with their respective characters.

**Fig.S11 Collinearity analysis of all detected collinear fragments among the 12 *Rosaceae* species.** Arcs connect homologous genomic regions, with red indicating sequences in reverse orientation and gray indicating sequences in collinear orientation.
