# Supplementary material for: Assembly and comparative analysis of the complete mitochondrial genomes of Pyrus calleryana Decne. and Pyrus betulifolia Bunge
Source: Front Plant Sci. 2026 Sep 21;17:1906069. doi: 10.3389/fpls.2026.1906069 (PMC13640305; doi:10.3389/fpls.2026.1906069)
Supplement: Supplementary file 2 [file SupplementaryFile1.zip › Suppl.all/Fig S.docx]

**Fig. S1 Coverage depth distribution for the mitogenome of *P. betulifolia*.** The estimated mean coverage depth is 224.16× based on second-generation sequencing data (A) and 472.91× based on Nanopore long-read sequencing data (B).

**Fig.S2 Coverage depth distribution for the mitogenome of *P. calleryana*.** The estimated mean coverage depth is 553.02x based on second-generation sequencing data (A) and 287.49× based on Nanopore long-read sequencing data (B).

**Fig.S3 Sequence alignment of the proteins encoded by the *atp1* and *nad2* genes in *P. betulifolia* and *P. calleryana*.** Numbers on the right indicate amino acid residue positions; identical residues are shown with conserved characters, while variable residues are displayed with their respective characters.

****Fig.S4 Distribution characteristics of repetitive elements in the**** mitogenomes ****of** *P. betulifolia* **and** *P. calleryana***.** (A) Abundance distribution of different SSR motif types in the two** *Pyrus* **species. (B) Distribution of SSR repeat frequencies within CDS, IGS, and introns. (C) Length distribution of dispersed repeats.**

**Fig.S5 The distribution of RNA editing sites among PCGs in the *P. betulifolia* and *P. calleryana* mitogenome.**

**Fig.S6 Nucleotide diversity analysis of 12 *Rosaceae* species.**
